# Mechanical Thrombectomy in Patients with Malignancy: Comparable Procedural Success but Less Favorable Long-Term Outcomes

**DOI:** 10.3390/brainsci16050526

**Published:** 2026-05-14

**Authors:** Sena Aksoy, Arsida Bajrami, Songül Şenadım, Serdar Geyik

**Affiliations:** 1 Department of Neurology, Istanbul Aydın University Florya Medical Park Hospital, Istanbul 34295, Turkey; 2 Department of Radiology, Istanbul Aydın University Florya Medical Park Hospital, Istanbul 34295, Turkey

**Keywords:** acute ischemic stroke, mechanical thrombectomy, malignancy, endovascular therapy

## Abstract

**Highlights:**

**What are the main findings?**

- Mechanical thrombectomy achieves similarly high procedural success and safety in patients with and without malignancy, including comparable reperfusion rates and hemorrhage risk.
- Despite comparable short-term outcomes, patients with malignancy have significantly worse long-term outcomes, including lower functional independence and higher 90-day mortality.

**What are the implications of the main findings?**

- Malignancy should not be considered a contraindication to mechanical thrombectomy, as technical efficacy and procedural safety are preserved.
- Treatment decisions should emphasize careful patient selection and multidisciplinary evaluation, as long-term prognosis is largely driven by the underlying cancer rather than the procedure itself.

**Abstract:**

**Background and Aims:** Patients with malignancy are frequently excluded from randomized thrombectomy trials, and evidence regarding the safety and efficacy of mechanical thrombectomy (MT) in this population remains incompletely defined. We aimed to compare procedural success, functional outcomes, and mortality between acute ischemic stroke (AIS) patients with and without malignancy undergoing MT. **Methods:** We retrospectively analyzed 110 patients treated with MT. Patients were stratified into two groups: those with malignancy (n = 48) and those without malignancy (n = 62). Baseline demographics, vascular risk factors, procedural metrics, angiographic outcomes, and clinical outcomes including functional independence (modified Rankin Scale [mRS] 0–2), 90-day mortality and intracranial hemorrhage were compared. **Results:** Baseline demographics and admission stroke severity were similar between groups. Smoking was significantly more frequent in the malignancy group (25% vs. 11.3%, *p* < 0.001). Successful reperfusion (TICI 2b-3) was achieved in 95.8% of malignancy patients and 98.4% of controls (*p* = 0.51). Functional independence at 90 days was lower in the malignancy group (42.6% vs. 61.3%, *p* = 0.04), whereas 90-day mortality was significantly higher (44.7% vs. 19.4%, *p* = 0.004); this increase in mortality remained significant after multivariate analysis. There were no significant differences in rates of intracranial hemorrhage between groups (*p* = 0.53). **Conclusions:** Mechanical thrombectomy is technically effective and safe in patients with malignancy; however, long-term functional recovery and survival are significantly worse, likely reflecting the effect of cancer itself rather than procedural factors. Careful patient selection and multidisciplinary decision-making are essential in this population.

## 1. Introduction

Mechanical thrombectomy has become the established standard of treatment for acute ischemic stroke. Multiple randomized controlled trials have demonstrated significant improvements in functional independence and survival with endovascular therapy. However, patients with malignancy were underrepresented or excluded from these trials, leaving uncertainty regarding the generalizability of these results to this population [1,2].

Cancer is present in approximately 5–10% of patients presenting with AIS, making it a clinically important comorbidity within the stroke population. Cancer promotes a prothrombotic state characterized by endothelial injury, chronic inflammation, and vascular toxicity related to oncologic therapies [3]. These interconnected mechanisms not only predispose patients to ischemic stroke but can also impair neurological recovery and worsen long-term outcomes.

Thrombolysis is often unsuitable for patients with cancer because of factors such as ongoing anticoagulation therapy, recent major surgery, intracranial metastases, or hematologic abnormalities, including coagulopathy and thrombocytopenia, leaving MT as the main reperfusion strategy for those who are eligible [3]. The available evidence on MT in patients with cancer comes solely from observational studies, case series, and retrospective analyses, all of which are inherently vulnerable to substantial selection bias and confounding [4,5,6,7]. Primary concerns about MT in patients with cancer include a potentially higher risk of hemorrhagic complications, distinct biological features of the thrombus, and the concern that advanced systemic illness may restrict the likelihood of achieving meaningful neurological recovery.

This study aims to compare procedural and clinical outcomes of MT in patients with and without malignancy.

## 2. Methods

### 2.1. Study Design and Population

This retrospective cohort study included patients admitted to our tertiary stroke center with a diagnosis of acute ischemic stroke and underwent mechanical thrombectomy. Patients were identified through institutional stroke databases and imaging archives. Patients were divided into two groups based on the presence of malignancy at the time of stroke presentation. Ethical approval for this study was obtained from Istanbul Aydın University Clinical Research Ethics Committee (Approval number: 133/2026, Date: 1 April 2026).

### 2.2. Data Collection

Demographic data, vascular risk factors, admission National Institutes of Health Stroke Scale (NIHSS) scores, time metrics from symptom onset to reperfusion, occlusion sites, endovascular techniques, and angiographic outcomes were recorded. Successful reperfusion was defined as a final TICI score of 2b-3. The choice of thrombectomy technique was determined by the neurointerventionalist based on vascular anatomy, thrombus characteristics, and procedural considerations. Both stent retriever and aspiration techniques were used, either alone or in combination, at the operator’s discretion. Procedures were generally performed under conscious sedation unless general anesthesia was required due to specific clinical conditions.

Clinical outcomes included NIHSS at 24 h and at discharge, modified Rankin Scale at discharge and 90 days, intracranial hemorrhage, and 90-day mortality. The causes of mortality in patients with malignancy were evaluated in two separate categories: those related to cancer and its associated conditions, and those attributable to stroke and stroke-related complications.

### 2.3. Statistical Analysis

SPSS Version 30.0.0 was used for statistical analysis. Continuous variables were expressed as mean ± standard deviation or median with interquartile range, depending on distribution, and were compared using Student’s *t*-test or Mann–Whitney U test as appropriate. Categorical variables were compared using the chi-square test or Fisher’s exact test. A *p*-value < 0.05 was considered statistically significant.

Binary logistic regression analyses were performed to evaluate factors associated with a favorable outcome (90-day mRS 0–2). Variables of clinical relevance were included in multivariable models. Potential confounding was assessed by comparing crude and adjusted odds ratios, and by calculating the percentage change in effect estimates.

## 3. Results

A total of 110 patients were included in the study. The malignancy group consisted of 48 patients, while 62 patients without malignancy served as controls. Baseline characteristics were largely similar between groups. There were no statistically significant differences in age, sex distribution, diabetes mellitus, hypertension, hyperlipidemia, atrial fibrillation, coronary artery disease, or prior stroke history (Table 1). Smoking was significantly more prevalent in patients with malignancy compared to controls (25% vs. 11.3%, *p* < 0.001). Admission NIHSS scores were similar between groups (14.9 ± 6 vs. 14.4 ± 6.8), indicating comparable stroke severity at presentation (*p* = 0.92, Table 1).

Breast cancer was identified in 6 (12.5%) patients, lung cancer in 21 (43.8%) patients, and gastrointestinal malignancies (gastric or colorectal cancer) in 10 (20.8%) patients, while the remaining 11 (22.9%) patients had other cancer types, including pancreatic, ovarian and hepatic malignancies. Within the malignancy group, 6 patients (12.5%) were classified as having non-metastatic disease, whereas 14 patients (29.2%) presented with metastatic cancer; data for the remaining patients were not available. With respect to disease activity status, 26 patients (54.2%) had active disease, and 5 patients (10.4%) were in remission, while information was missing for 17 patients (35.4%).

In the malignancy group, arterial occlusion was identified in the internal carotid artery (ICA) in 9 (18.8%) patients, in M1 segment of the middle cerebral artery (MCA) in 15 (31.3%), in MCA M2 segment in 10 (20.8%) patients, and in distal vessels in 3 (6.3%) patients (Table 2). Similarly, in the non-malignancy group, occlusion was detected in the ICA in 13 (21%) patients, in the M1 segment in 25 (40.3%) patients, in the M2 segment in 14 (22.6%) patients and in distal vessels in 2 (3.2%) patients. Tandem occlusion was observed in 10 (20.8%) patients with malignancy and in 3 (4.8%) patients without malignancy (*p* = 0.01). Basilar artery occlusion was identified more frequently among patients without malignancy (5, 8.1%) compared to those with malignancy, but it was not statistically significant (1, 2.1%, *p* = 0.26, Table 2).

Procedural metrics, including onset-to-door time, door-to-puncture time, and puncture-to-recanalization time, did not differ significantly between groups (Table 2). Successful reperfusion (TICI 2b-3) was achieved in 95.8% of patients in the malignancy group and 98.4% of controls, demonstrating similarly high technical success rates (*p* = 0.51). First-pass effect and total number of thrombectomy passes were also comparable (*p* = 0.2 and *p* = 0.87, respectively).

**Figure 1:**
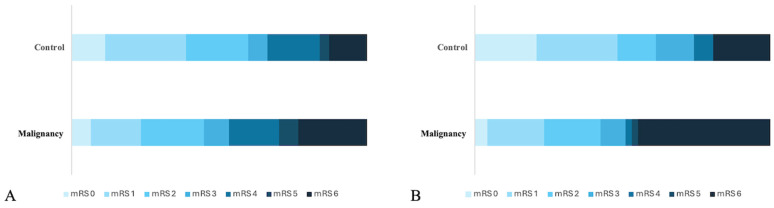
(**A**) Discharge mRS scores of the patients with and without malignancy. (**B**) 90-day mRS scores of the same patient cohorts.

Early neurological outcomes, as assessed by NIHSS at 24 h and at discharge, did not differ significantly between groups (*p* = 0.51, *p* = 0.1). Similarly, functional independence at discharge -mRS of 0–2- was not significantly different between the two groups (*p* = 0.87, Table 2, Figure 1A). In-hospital mortality occurred in 11 (22.9%) patients with malignancy and in 8 (12.9%) patients without malignancy (*p* = 0.13, Figure 1A). The incidence of intracranial hemorrhage was similar (41.3% in the malignancy group vs. 35.5% in controls, *p* = 0.53).

However, significant differences emerged in long-term outcomes. Functional independence at 90 days was achieved in 42.6% of patients with malignancy compared to 61.3% of controls (*p* = 0.04, Table 2, Figure 1B). Furthermore, 90-day mortality was significantly higher in the malignancy group (44.7% vs. 19.4%, *p* = 0.004, Figure 1B). In subgroup analysis, in patients in remission, no mortality was observed at 90 days, and all patients were functionally independent (*p* = 0.048, *p* = 0.015). Similarly, the non-metastatic patients demonstrated lower mortality alongside a higher level of functional independence, but this difference did not reach statistical significance (*p* = 0.1, *p* = 0.8, Table 3).

In multivariable logistic regression analyses adjusting for age, baseline NIHSS, and onset-to-door time, malignancy was associated with a lower likelihood of a favorable outcome (90-day mRS 0–2), although this did not reach statistical significance (OR: 0.39, 95% CI: 0.15–1.05, *p* = 0.055). In contrast, malignancy was independently associated with increased mortality (OR: 3.75, 95% CI: 1.33–10.53, *p* = 0.007, Table 4). Higher NIHSS scores and longer onset-to-door times were independently associated with a worse favorable outcome. After further adjustment for recanalization status, the association between malignancy and favorable outcome remained non-significant (OR: 0.40, *p*: 0.067), whereas malignancy continued to be independently associated with increased mortality (OR: 3.87, *p*: 0.005, Table 4).

To quantify the potential confounding effect of tandem occlusion, crude and adjusted odds ratios for the association between malignancy and favorable outcome (90-day mRS 0–2) were compared using logistic regression analysis. The inclusion of tandem occlusion in the multivariable model resulted in only a 0.3% change in the effect estimate, indicating no evidence of meaningful confounding. However, given the limited number of patients with tandem occlusion (n = 13), these findings may be underpowered and should be interpreted accordingly.

Among patients with malignancy, analysis of the causes of death revealed that 9 (42.9%) patients died due to ischemic stroke and its related complications, whereas 12 (57.1%) patients died from cancer and cancer-associated conditions.

## 4. Discussion

This study showed that mechanical thrombectomy in patients with malignancy yields technical and procedural results similar to those seen in individuals without cancer. Rates of successful recanalization and the number of passes were not negatively influenced by the presence of malignancy. Similarly, procedural safety -evidenced by comparable rates of intracranial hemorrhage- remained unaffected. Together, these results indicate that, from both a technical and periprocedural perspective, mechanical thrombectomy is a feasible and effective treatment option in this population.

Our findings are consistent with prior observational studies demonstrating that the feasibility and angiographic efficacy of mechanical thrombectomy (MT) are preserved in patients with acute ischemic stroke and malignancy [1,2]. Reported reperfusion rates are comparable to those in non-cancer populations, supporting the view that malignancy should not be considered a contraindication to MT. In our cohort, the overall rate of intracranial hemorrhage was 41.3% in the malignancy group and 35.5% in controls, with no statistically significant difference between groups. Symptomatic intracranial hemorrhage rates were also comparable (10.4% vs. 4.8%). Although the overall hemorrhage rate in our cohort appears somewhat higher than in some previously reported series, this likely reflects broader definitions of hemorrhagic transformation, including asymptomatic subtypes. We did not observe an increased risk of symptomatic intracranial hemorrhage, in line with literature showing that cancer is not an independent predictor of hemorrhagic transformation after MT [5,8]. Together, these data challenge earlier assumptions that cancer patients are inherently at higher risk of hemorrhage or unlikely to benefit, and support offering reperfusion therapy when clinically appropriate.

Although short-term outcomes were comparable between groups, long-term outcomes remained significantly worse in patients with malignancy. This divergence indicates that determinants of prognosis extend beyond technical reperfusion and early neurological recovery. Similar patterns have been described across multiple cohorts, underscoring that even when early neurological improvement is achieved and short-term recovery appears favorable, the durability of functional independence is often limited in patients with underlying malignancy [8,9,10,11].

In this study, malignancy was not independently associated with a 90-day favorable functional outcome but was strongly associated with increased mortality in patients undergoing thrombectomy. The lack of a significant association between malignancy and a favorable functional outcome may be explained by the small sample size and limited statistical power.

In contrast, malignancy emerged as a strong and independent predictor of 90-day mortality. The substantially higher 90-day mortality observed in our cohort further reinforces the notion that an acute ischemic event occurring in the context of cancer frequently reflects an advanced and complex systemic condition rather than an isolated vascular insult. Given the comparable early neurological courses between groups, the excess mortality is unlikely to be attributable solely to stroke-related mechanisms or procedural factors. Instead, the cumulative burden of systemic disease, cancer-related complications, prothrombotic states, treatment-related effects, and competing oncologic mortality likely play pivotal roles in shaping long-term outcomes. Previous studies have shown that mechanical thrombectomy in patients with malignancy is associated with comparable rates of hemorrhagic complications and technical success compared to non-cancer patients. However, poorer survival observed in this population appears to be largely driven by underlying cancer-related factors rather than differences in stroke severity or procedural outcomes [12]. Collectively, these findings emphasize that while mechanical thrombectomy can achieve similar technical success, overall prognosis in patients with malignancy is largely determined by the underlying systemic disease trajectory.

Although malignancy was associated with worse 90-day outcomes, this relationship should be considered with caution. Other factors, including differences in post-stroke care, comorbidities, and overall clinical condition, may also contribute to the observed outcomes. Therefore, attributing poorer long-term outcomes solely to malignancy may oversimplify a multifactorial process.

In our cohort, the proportion of tandem occlusions was higher in patients with malignancy, raising the possibility that this imbalance could contribute to worse outcomes. Given the known association of tandem occlusions with increased procedural complexity and poorer functional recovery, we specifically evaluated their potential confounding effect. However, our sensitivity analysis demonstrated that the inclusion of tandem occlusion in the multivariable model did not significantly change the effect estimate for malignancy, suggesting that tandem occlusion is unlikely to be a major driver of the observed outcomes. Nonetheless, this finding should be viewed considering the limited number of tandem occlusion cases, which may reduce the statistical power to detect a modest effect.

In subgroup analyses, patients with active disease and those with metastatic cancer tended to have higher mortality and lower rates of functional independence compared to patients in remission or without metastasis. However, these findings should be interpreted with caution due to the very small sample sizes within these subgroups, which substantially limit statistical power and preclude definitive conclusions.

As growing data have demonstrated that MT is both feasible and safe in cancer patients -including those with metastatic disease- the number of metastatic cancer patients undergoing MT has increased compared with earlier periods, when malignancy was frequently regarded as a relative contraindication [13]. As collective experience with mechanical thrombectomy in patients with malignancy continues to grow, patient selection is expected to become increasingly refined and precise, enabling clinicians to better identify those most likely to get meaningful clinical benefit.

These findings highlight the need for individualized, multidisciplinary decision-making when considering mechanical thrombectomy (MT) in patients with cancer. Contemporary evidence supports a collaborative approach involving neurologists, neurointerventionalists, and oncologists, with treatment decisions guided by overall prognosis, anticipated survival, and quality of life rather than the presence of malignancy. Future studies incorporating detailed oncologic characteristics are needed to better define which cancer patients may benefit most from mechanical thrombectomy.

This study has several limitations. First, it is retrospective and conducted at a single center, which may limit the generalizability of the findings. One of the most important limitations is the relatively small sample size and the wide confidence intervals, which restrict the ability to perform robust multivariable adjustments and detailed subgroup analyses. Additionally, potential residual confounding and selection bias cannot be excluded. Patients with better overall clinical status may have been preferentially selected for treatment, introducing potential selection bias. Finally, detailed oncologic characteristics -such as cancer type, stage, metastatic burden, and treatment regimens were not evaluated, although these factors may substantially influence both patient selection and treatment outcomes. In particular, the absence of stratification by cancer stage (e.g., limited-stage versus metastatic disease) limits the interpretability of the observed associations and precludes translating our findings into clinically meaningful selection guidance. 

## 5. Conclusions

Mechanical thrombectomy appears to achieve similar angiographic and procedural success in patients with and without malignancy. However, the lower rates of functional independence and higher 90-day mortality observed in patients with cancer suggest that longer-term outcomes may be influenced more by the underlying disease than by recanalization alone. These findings support considering thrombectomy in carefully selected cancer patients, while highlighting the need for individualized, multidisciplinary assessment of prognosis and treatment goals. However, the heterogeneity of the malignancy population, particularly the lack of detailed stratification by cancer stage and prognosis, limits the direct applicability of these findings to clinical decision-making. Further prospective studies with larger cohorts are warranted to better define patient selection and improve outcome prediction in this complex and increasingly encountered population.

## Figures and Tables

**Table 1:** Baseline characteristics of the patients.

	Malignancy (n = 48)	Control (n = 62)	*p* Value
**Demographics**			
Age (mean ± SD)	66.1 ± 10	69.7 ± 10.8	0.11
Female sex (n, %)	21 (43.8)	24 (38.7)	0.59
**Medical history**			
Diabetes (n, %)	17 (35.4)	24 (38.7)	0.78
Hypertension (n, %)	35 (72.9)	36 (58.1)	0.07
Hyperlipidemia (n, %)	22 (45.8)	22 (35.5)	0.23
Atrial fibrillation (n, %)	10 (20.8)	22 (35.5)	0.1
CAD (n, %)	14 (29.2)	12 (19.4)	0.2
Previous stroke (n, %)	7 (14.6)	5 (8.1)	0.25
Smoking (n, %)	12 (25)	7 (11.3)	<0.001 *
**Clinical features**			
NIHSS on admission (mean ± SD)	14.9 ± 6	14.4 ± 6.8	0.92
Onset-to-door (min, mean ± SD)	249.05 ± 163.2	227.5 ± 173.6	0.38
Door-to-puncture (min, mean ± SD)	61.2 ± 31.9	62.4 ± 25.3	0.57
Puncture-to-recanalization (min, median, IQR)	32.5 (17–59.25)	30 (16.5–43.25)	0.75

* *p* < 0.05.

**Table 2:** Clinical outcome and endovascular procedural metrics.

	Malignancy (n = 48)	Control (n = 62)	*p* Value
**Outcome parameters**			
NIHSS 24th hours (mean ± SD)	10.4 ± 7.5	7.7 ± 7.5	0.51
NIHSS discharge (mean ± SD)	6.8 ± 5.1	5.3 ± 5.6	0.1
Any intracranial hemorrhage (n, %)	19 (41.3)	22 (35.5)	0.53
HT1 (n, %)	6 (12.5)	9 (14.5)	0.53
HT2 (n, %)	2 (4.2)	4 (6.5)	0.49
PH1 (n, %)	5 (10.4)	2 (3.2)	0.17
PH2 (n, %)	4 (8.3)	4 (6.5)	0.47
SAH (n, %)	4 (8.3)	3 (4.8)	0.34
sICH (n, %)	5 (10.4)	3 (4.8)	0.23
mRS 0–2 discharge (n, %)	21 (44.7)	37 (59.7)	0.87
Mortality discharge	11 (22.9)	8 (12.9)	0.13
mRS 0–2 90-day (n, %)	20 (42.6)	38 (61.3)	0.04 *
Mortality 90-day (n, %)	21 (44.7)	12 (19.4)	0.004 *
**Occlusion sites**			
ICA (n, %)	9 (18.8)	13 (21)	0.4
M1 (n, %)	15 (31.3)	25 (40.3)	0.22
M2 (n, %)	10 (20.8)	14 (22.6)	0.65
Distal (n, %)	3 (6.3)	2 (3.2)	0.38
Tandem (n, %)	10 (20.8)	3 (4.8)	0.01 *
Basilar (n, %)	1 (2.1)	5 (8.1)	0.26
**Endovascular treatment**			
Stentretriever (n, %)	39 (81.3)	46 (74.2)	0.21
First pass effect (n, %)	32 (66.7)	48 (77.4)	0.2
Final TICI 2b-3 (n, %)	46 (95.8)	61 (98.4)	0.51
Total number of pass (mean ± SD)	1.8 ± 1.2	1.7 ± 1	0.87

* *p* < 0.05.

**Table 3:** 90-day outcomes according to remission status and presence of metastasis.

	Mortality	Functional Independence
Remission status		
Active disease	13 (13/26)	7 (7/26)
Remission	0 (0/5)	5 (5/5)
**Presence of metastasis**		
Metastatic	8 (8/14)	3 (3/14)
Non-metastatic	1 (1/6)	4 (4/6)

**Table 4:** Multivariable logistic regression analyses for favorable functional outcome and mortality.

Variable	OR	95% CI	*p* Value
Favorable functional outcome (90-day mRS 0–2): multivariable model without recanalization			
Malignancy	0.39	0.15–1.05	0.055
Age (per year)	1.04	0.99–1.09	0.072
NIHSS (per point)	0.995	0.992–0.998	0.002 *
Onset-to-door time	0.85	0.76–0.93	<0.001 *
**Favorable functional outcome (90-day mRS 0–2): multivariable model with recanalization**			
Malignancy	0.4	0.15–1.05	0.067
Age (per year)	1.05	1.00–1.10	0.067
NIHSS (per point)	0.995	0.992–0.998	0.002 *
Onset-to-door time	0.85	0.76–0.93	<0.001 *
Recanalization	2.75	0.30–25.3	0.423
**Mortality: multivariable model without recanalization**			
Malignancy	3.75	1.33–10.53	0.007 *
Age (per year)	1.02	0.98–1.07	0.383
NIHSS (per point)	1.002	0.999–1.005	0.117
Onset-to-door time	1.08	0.99–1.17	0.063
**Mortality: multivariable model with recanalization**			
Malignancy	3.87	1.38–10.85	0.005 *
Age (per year)	1.02	0.98–1.07	0.373
NIHSS (per point)	1.002	0.999–1.005	0.115
Onset-to-door time	1.07	0.99–1.16	0.075
Recanalization	0.22	0.002–3.61	0.235

* *p* < 0.05. OR: odds ratio; CI: confidence interval.

## Data Availability

All data generated or analyzed during this study are included in this article. Further enquiries can be directed to the corresponding author.
